# Understanding the “individual drug reaction” from the perspective of the interaction between probiotics and lovastatin in vitro and in vivo

**DOI:** 10.1186/s40168-023-01658-z

**Published:** 2023-09-25

**Authors:** Siyuan Shen, Jun Wang, Chenchen Ma, Yanni Chen, Hao Ding, Jiachao Zhang

**Affiliations:** 1 School of Food Science and Engineering, Hainan University, Haikou, China; 2 One Health Institute, Hainan University, Haikou, 570228 Hainan China; 3 Key Laboratory of Food Nutrition and Functional Food of Hainan Province, Haikou, 570228 China

**Keywords:** Probiotics, Lovastatin, Gut microbiota, *Lactiplantibacillus plantarum*, *Lacticaseibacillus paracasei* strain Shirota

## Abstract

**Background:**

The existence of the gut microbiota produces an “individual drug reaction.” As members of the intestinal microbiota, probiotics, although they have prebiotic functions, may accelerate the degradation of drugs, thereby affecting drug efficacy. Lovastatin is one of the well-recognized lipid-lowering drugs. Its main action site is the liver. Therefore, if it is degraded in advance by gastrointestinal probiotics, its efficacy may be reduced.

**Results:**

Here, we designed a two-stage experiment in vitro and in vivo to explore the degradation of lovastatin by probiotics. In vitro, the degradation of lovastatin by 83 strains of *Lactiplantibacillus plantarum* and the “star strain” *Lacticaseibacillus paracasei* strain Shirota was investigated by high-performance liquid chromatography (HPLC). The results showed that probiotics could degrade lovastatin to varying degrees. Subsequently, we selected *Lactiplantibacillus plantarum* A5 (16.87%) with the strongest ability to degrade lovastatin, *Lactiplantibacillus plantarum* C3 (4.61%) with the weakest ability to degrade lovastatin and *Lacticaseibacillus paracasei* strain Shirota (17.6%) as representative probiotics for in vivo experiments. In vivo, the therapeutic effect of lovastatin combined with probiotics on golden hamsters with mixed hyperlipidemia was evaluated by measuring blood indicators, intestinal microbiota metagenomic sequencing, and the liver transcriptome. The results showed that the intake of probiotics did not affect the efficacy of lovastatin and could slow the inflammatory reaction of the liver.

**Conclusions:**

The supplementation of probiotics produced beneficial metabolites in the intestine by promoting beneficial microbes. Intestinal metabolites affected the expression of the liver genes through the gut-liver axis, increased the relative content of the essential amino acids, and finally improved the liver inflammatory response of the host. This study aims to reveal the impact of probiotics on the human body from a unique perspective, suggesting the impact of taking probiotics while taking drugs.

Video Abstract

**Supplementary Information:**

The online version contains supplementary material available at 10.1186/s40168-023-01658-z.

## Background

Hundreds of different kinds of bacterial communities, collectively known as the intestinal microbiome, inhabit the human intestine [1]. They are closely related to human health and disease and vary from individual to individual. Some people take the same drug with obvious effects, while some people take it with no effect and even have side effects. This “individual drug reaction” phenomenon was reported to be related to the human gut microbes [2]. Recently, scientists have begun to explore the systematic map of the interaction between drugs and bacteria [3] and have found that microorganisms can change the activity and efficacy of drugs through chemical transformation of drugs [4]. Probiotics are considered to be intestinal microorganisms with probiotic functions, such as improving immunity and maintaining the structural balance of the gut microbiota [5, 6]. An increasing number of people choose to consume probiotics to remain healthy [7]. However, there are two sides to everything. Whether taking probiotics while taking drugs still has a probiotic effect on the body or whether the intake of probiotics will accelerate the chemical transformation of drugs and thereby affect the role of drugs at specific locations and thus reduce the efficacy is worth exploring.

By consulting the literature, we inferred that the effect of probiotics on the treatment of high cholesterol by lovastatin may not be beneficial because of the action site and mechanism of activity of lovastatin, and probiotics may reduce the efficacy of lovastatin. Lovastatin is one of the most widely used lipid-lowering drugs and is commonly used to treat hypercholesterolaemia and mixed hyperlipidaemia [8]. Lovastatin is an inactive prodrug that needs to be hydrolyzed into the active hydroxy acid lovastatin in vivo to play its role [9]. Lovastatin, an active hydroxy acid, inhibits the synthesis of cholesterol by inhibiting 3-hydroxy-3-methyl glutaryl coenzyme A reductase (HMG-CoA), the key enzyme in the synthesis of cholesterol, to exert its efficacy [10]. However, HMG-CoA is located on the membrane of human and animal liver cells [11], so the main site of action of lovastatin is the liver. Oral lovastatin plays a role in the liver through the oesophagus, gastrointestinal tract, and then to the liver. Consequently, if lovastatin is degraded into active lovastatin hydroxy acid by intestinal probiotics when it reaches the gastrointestinal tract and plays an active role, lovastatin reaching the liver will be reduced, thus affecting its efficacy. Consequently, based on the above understanding of the lovastatin treatment mechanism, we are eager to explore the following three key issues. The first question is whether probiotics can degrade lovastatin. The second question is whether probiotics affect the efficacy of lovastatin. The third question is whether the synergistic intake of lovastatin and probiotics will affect other organs of the body, and if so, what is its mechanism of action?

Therefore, we explored the above three key issues based on the current lack of research on probiotic degradation of drugs. Aiming at the hypothesis that the degradation of lovastatin by probiotics will reduce its efficacy, 83 strains of *Lactiplantibacillus plantarum* [12] from the self-built bacteria bank and the “star strain” *Lacticaseibacillus paracasei* strain Shirota [13] were taken as the research objects, and two-stage experiments in vitro and in vivo were designed. The degradation effect of probiotics on lovastatin was preliminarily evaluated by high-performance liquid chromatography (HPLC) in vitro. At the same time, we monitored the transcriptional changes in probiotics at different time points under the action of lovastatin and explored the possible reasons for the degradation of lovastatin by probiotics. Subsequently, in vivo experiments confirmed the therapeutic effect of lovastatin and probiotics on mixed hyperlipidaemia in golden hamsters. The influence of probiotics on lovastatin treatment was further discussed based on the characterization results, intestinal microbiota metagenomic sequencing, intestinal metabolites, and liver transcription. This study aims to reveal the impact of probiotics on the human body from a unique perspective, suggesting the impact of taking probiotics while taking drugs.

## Methods

### In vitro exploration experiment

#### Probiotics and drug

All 83 probiotic *Lactiplantibacillus plantarum* strains used in the experiment were provided by the tropical probiotic lactobacillus species Bank of Hainan University. The experimental drug lovastatin (pharmaceutical secondary standard, certified reference material) was purchased from Sigma Aldrich, Inc.

#### Experimental design

Lovastatin alone was cultured in MRS at 37 ℃, away from light. At the same time, lovastatin was respectively co-cultured with 83 strains of *Lactiplantibacillus plantarum* and a strain of *Lacticaseibacillus paracasei* strain Shirota and cultured in MRS medium for 6 h and 12 h (away from light, 37 ℃). The detailed procedure of in vitro experiment has been shown in the flow chart (Supplemental Fig. 1). The final added concentration of lovastatin was 33 μM, which was consistent with the estimation of gastrointestinal drug concentration [14, 15]. Subsequently, the supernatant culture solution was analyzed by the HPLC of Agilent Technologies to obtain the response peak area of lovastatin [16] (column: C-18 Hypersil column; column temperature: 30 ℃; mobile phase: acetonitrile + 0.1% orthophosphate:water + 0.1% orthophosphate = 80:20; flow rate: 1.5 ml/min detection: 238 nm UV detection). In addition, the peak area of lovastatin cultured with synergistic probiotics was compared with that cultured with lovastatin alone, and the degradation percentage of lovastatin by probiotics was calculated.

The calculation formula of lovastatin degradation percentage is as follows:

$$\mathrm{V }=\frac{\mathrm{A}-\mathrm{B}}{\mathrm{A}}$$

V: Percentage of lovastatin degradation by probiotics

A: Peak area of lovastatin

B: Peak area of lovastatin after synergistic probiotic culture

At the same time, the transcriptome (Beijing Novogene Technology Co., Ltd.) of three probiotics (cultured alone and simultaneously with lovastatin) at six time points (0 h, 3 h, 6 h, 12 h, 24 h, and 36 h) was determined to explore the possible reasons for the degradation of lovastatin by probiotics.

### In vivo experiment

#### Animals

This study was approved by the Animal Ethics Committee of Hainan University, and all animal operations were carried out in accordance with the “Guidelines for The Care and Use of Experimental Animals” of Hainan University. Thirty-six male-specific pathogen-free (SPF) golden hamsters were purchased from Beijing Vital River Laboratory Animal Technology Co., Ltd. (5 weeks of age). Golden hamsters were allowed to acclimatize for a week before the experiment and were fed a sterilized feed and water under SPF conditions. The food feeds and bedding materials of the control group and the mixed hyperlipidemia model group were provided by Jiangsu Syony Pharmaceutical Bioengineering, Co., LTD. The control group was fed with maintenance base feed (grain raw material 80%, animal protein 10%, small feed additive 10%), and the model group was fed with 45% fat + 0.5% cholesterol energy feed (maintenance base 43%, lard 17.5%, sucrose 12%, whole milk powder 10%, casein 13%, experimental animal premix 2%, calcium hydrogen phosphate 2%, 0.5% cholesterol).

#### Experimental design

In the experiment, the golden hamsters were randomly divided into 6 groups (*n* = 6), which were respectively: blank control group (control, Ctrl) fed with maintenance base diet, hyperlipidemia model group (model + carboxymethyl cellulose, MO), hyperlipidemia lovastatin treatment group (model + lovastatin, Lov), lovastatin combined with *Lactiplantibacillus plantarum* A5 to treat hyperlipidemia model group (model + lovastatin + *Lactiplantibacillus plantarum* A5, L + A5), lovastatin combined with *Lactiplantibacillus plantarum* C3 to treat hyperlipidemia model group (model + lovastatin + *Lactiplantibacillus plantarum* C3, L + C3), and lovastatin combined with *Lacticaseibacillus paracasei* strain Shirota to treat hyperlipidemia model group (model + lovastatin + *Lacticaseibacillus paracasei* strain Shirota, L + LcS). The model groups were fed high-fat diet for 8 weeks, and the mixed hyperlipidemia model was established. Then the orbital blood was taken, and the model groups were compared with the blank control group to confirm the results of modeling.

Then, the model groups were treated for 4 weeks. The MO group was given carboxymethyl cellulose (0.5 mL) daily by gavage; the Lov group was given 5 mg/kg lovastatin daily (dissolved with carboxymethyl cellulose, 0.5 mL); In the L + A5 group, 5 mg/kg lovastatin + 10^8^ cfu *Lactiplantibacillus plantarum* A5 (dissolved with carboxymethyl cellulose, 0.5 mL) was administered daily; In group L + C3, 5 mg/kg lovastatin + 10^8^ cfu *Lactiplantibacillus plantarum* C3 (dissolved with carboxymethyl cellulose, 0.5 mL) was administered daily; the L + LcS group was given 5 mg/kg lovastatin + 10^8^ cfu *Lacticaseibacillus paracasei* strain Shirota (dissolved with carboxymethyl cellulose, 0.5 mL) daily. After 4 weeks of treatment, judge the influence of the presence of probiotics on the therapeutic effect of lovastatin.

#### Sample collection and measurement

Feces, serum, and tissue samples of golden hamsters were collected under sterile conditions. Feces were collected at weeks 8 and 12, before which each hamster was allowed to excrete overnight in a newly cleaned cage. The golden hamsters were anesthetized, and after their reflexes disappeared, the blood was taken from their orbits. The blood was then coagulated at room temperature for 20 min and centrifuged for 20 min (3000 rpm). Carefully collect the supernatant, and the supernatant is the serum sample. Fecal and serum samples were kept at − 80 ℃ until use. Feces were used for the determination of metagenome (Beijing Novogene Technology Co., Ltd.), and serum samples were used for the determination of basic blood indicators (ELISA kit, Xin Yu Biotechnology Co., Ltd, Shanghai, China). Once the orbital blood has been taken, we will execute the golden hamster with a short neck. The golden hamsters were then displaned, and the liver tissue and colon contents were carefully removed. The liver tissue is divided into two parts: one part is washed with 0.85% normal saline and fixed in paraformaldehyde solution and then used for the determination of liver tissue sections (Wuhan Saville Biotechnology Co., Ltd.). In the other part, it was quickly frozen with liquid nitrogen and stored at − 80 ℃ for the determination of liver transcriptome (Beijing Novogene Technology Co., Ltd.). Colon contents were stored at − 80 ℃ for the determination of SCFAs. The intestinal contents were dissolved with saturated NaCl for 30 min and homogenized and then acidified with sulfuric acid. After acidification, the supernatant was extracted with ether for 30 min. The supernatant was centrifuged and put into a gas phase sampling bottle for determination. Gas chromatography mass spectrometry (GC–MS) determination of short-chain fatty acids by Agilent Technology Co., Ltd. [17]. (column: Agilent DB-WAX, 0.25 mm × 0.25μ m × 50 cm; injection port temperature: 250 ℃; gas interface temperature: 250° C; carrier gas flow rate: 1.5 mL/min; split ratio, 3:1; injection volume: 1 μL).

#### Quality control and data processing of high throughput metagenomic sequencing

All fecal DNA samples were sequenced in the same batch using Illumina HiSeq 2500 platform. The data used for analysis is screened by strict standards. When *N* contents read at any time exceed 10% of the reading base, paired reads will be deleted. When the number of low-quality bases (Q ≤ 5) in any read operation exceeds 50%, the paired read operation is cancelled. On average, each sample obtained 11.07 GB of high-quality paired end sequencing data, and a total of 3189.48 GB of high-quality data were obtained in this project (online Supplementary Table S1).

For metagenomics species annotation, Kraken2 + Bracken software were used. Kraken2 classifies metagenome sequences with high accuracy, and Bracken calculates species abundance in metagenome data [18]. Subsequently, false positives with species abundance < 0.01% were removed.

#### RNA sequencing and transcriptome analysis

For analysis of transcriptome data, Illumina NovaSeq 6000 was sequenced using standard protocols after RNA libraries were constructed. The screened reads were mapped to the reference genome using a STAR comparator [19]. Featurests were used to estimate gene expression [20]. Differential expression analysis of gene centers was performed using Bioconductor package DESeq2.

#### Statistics statement

All statistical analyses were performed using R (version 3.6.1) software. Boxplot, fit smooth, the bubble diagram, and bar charts were shown by the “ggplot2” package. PCoA analysis was performed using the “ade4” package in R [21]. Heatmaps were constructed using the “pheatmap” package. The Vioplot was built using the “vioplot” package. The networks were calculated using the Spearman’s rank correlation coefficient and were visualized in Cytoscape (version 3.4). The differential abundances of genera and species were identified with the Wilcoxon rank-sum test [22] and were considered the significance according to the *P* value threshold at 0.05 (*p < 0.05, **p < 0.01). The Metascape was used for enrichment analysis of differential genes (http://metascape.org). Volcano plots were built using an online tool called “Omicstudio.” The illustration of experimental designs and the potential biological mechanisms uncovered by our study were constructed by using “BioRender.”

## Results

### Probiotics exhibited strain specificity with respect to lovastatin degradation in vitro

**Fig. 1:**
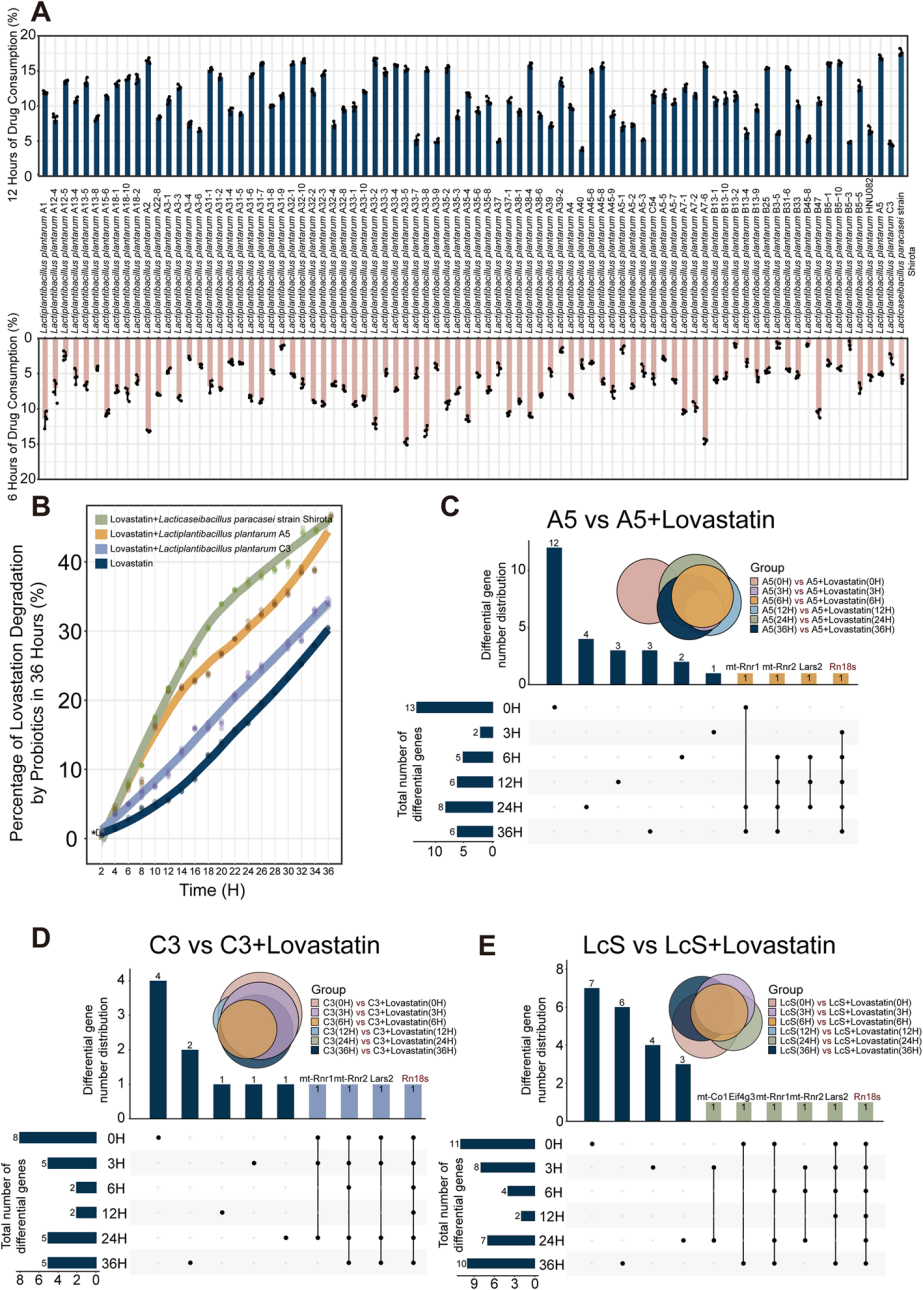
Probiotics exhibited different degrees of degradation of lovastatin in vitro.** A** Percentage consumed at 6 and 12 h after lovastatin was incubated with 83 strains of *Lactiplantibacillus plantarum* and one strain of *Lacticaseibacillus paracasei* strain Shirota. Bar charts and error bar charts represented the mean and standard error of *n* = 4 test repetitions. **B** Pharmacokinetics within 36 h: lovastatin cooperated with three representative probiotic strains and lovastatin (*p < 0.05). **C** The transcriptome differential gene expression of *Lactiplantibacillus plantarum* A5 and *Lactiplantibacillus plantarum* A5 incubated with lovastatin at 6 time points (0 h, 3 h, 6 h, 12 h, 24 h, and 36 h). **D** The transcriptome differential gene expression of *Lactiplantibacillus plantarum* C3 and *Lactiplantibacillus plantarum* C3 incubated with lovastatin at 6 time points (0 h, 3 h, 6 h, 12 h, 24 h, and 36 h). **E** The transcriptome differential gene expression of *Lacticaseibacillus paracasei* strain Shirota and *Lacticaseibacillus paracasei* strain Shirota incubated with lovastatin at 6 time points (0 h, 3 h, 6 h, 12 h, 24 h, and 36 h)

We measured the percentage of lovastatin consumed after incubation with 83 strains of *Lactiplantibacillus plantarum* and the “star strain” *Lacticaseibacillus paracasei* strain Shirota for 6 h and 12 h. After 12 h of incubation, 17.6% of lovastatin was degraded by *Lacticaseibacillus paracasei* strain Shirota. In addition, *Lactiplantibacillus plantarum* A5 showed the strongest ability to degrade lovastatin (16.87%), and *Lactiplantibacillus plantarum* C3 showed the weakest ability (4.61%) (Fig. 1A). The above three strains were selected as representative probiotics. The pharmacokinetics after 36 h of construction showed that the drug degradation rates of the three probiotics were significantly higher than those of lovastatin alone after 2 h of incubation (*p* < 0.05) (Fig. 1B). It is suggested that probiotics can degrade lovastatin in vitro.

To explore how probiotics consume lovastatin, the transcription of genes related to this process was analyzed. Under the condition of cotreatment with lovastatin, the three probiotics showed highly similar differential transcription. Compared with the transcription of *Lactiplantibacillus plantarum* A5 itself, lovastatin coincubation with *Lactiplantibacillus plantarum* A5 produced 13, 2, 5, 6, 8, and 6 differentially expressed genes at 0, 3, 6, 12, 24, and 36 h, respectively (|Log2FoldChange|≥ 1, *p* < 0.05). Among them, the differentially expressed mt-Rnr1, mt-Rnr2, and Lars2 genes were the same at some time points, and the Rn18s gene was differentially expressed at five time points except at 0 h (Fig. 1C). Subsequently, *Lactiplantibacillus plantarum* C3, in collaboration with lovastatin, produced 8, 5, 2, 2, 5, and 5 differentially expressed genes at 0, 3, 6, 12, 24, and 36 h, respectively (|Log2FoldChange|≥ 1, *p* < 0.05). At some time points, mt-Rnr1, mt-Rnr2, and Lars2 were all differentially expressed. The Rn18s gene was different at 6 time points (Fig. 1D). At the same time, in combination with lovastatin, *Lacticaseibacillus paracasei* strain Shirota produced 11, 8, 4, 2, 7, and 10 differentially expressed genes at 0, 3, 6, 12, 24, and 36 h (|Log2FoldChange|≥ 1, *p* < 0.05). At some time points, mt-Co1, Eif4g3, mt-Rnr1, mt-Rnr2, and Lars2 were all differentially expressed. Similarly, the Rn18s gene was different at 6 time points (Fig. 1E). The transcriptional results showed that the presence of lovastatin reduced the expression of the Rn18s gene in three probiotics.

### Synergistic intake of probiotics did not affect the therapeutic effect of lovastatin against hyperlipidaemia, but was beneficial for liver protection in vivo

**Fig. 2:**
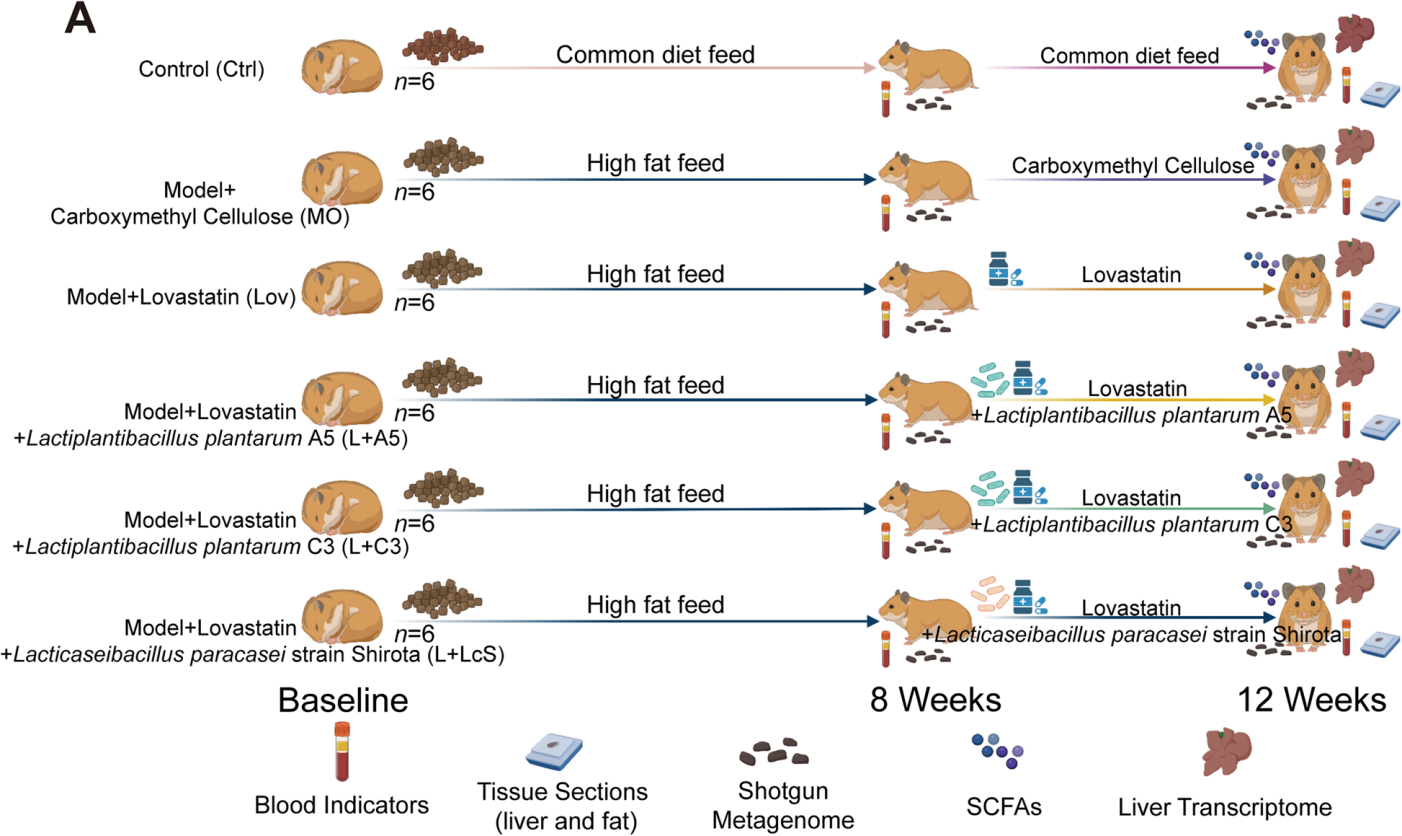
Effect of synergistic probiotics on lovastatin in vitro experimental design.** A** Schematic diagram showing experimental design. The date of constructing mixed hyperlipidemia model was defined as the baseline, on which golden hamsters were fed with high-fat diet. Lovastatin, lovastatin plus probiotics were administered daily from week 8 to week 12 (Lov group, L + A5 group, L + C3 group, and L + LcS group), and the MO group was replaced by carboxymethyl cellulose. The golden hamsters were killed in the 12th week. At the 8th week and the 12th week, fecal samples were collected for shotgun metagenomic sequencing, and serum was collected for blood index detection. At the 12th week, liver tissues were collected for the determination of liver transcriptome and liver tissue sections, and intestinal contents were collected for the determination of SCFAs

**Fig. 3:**
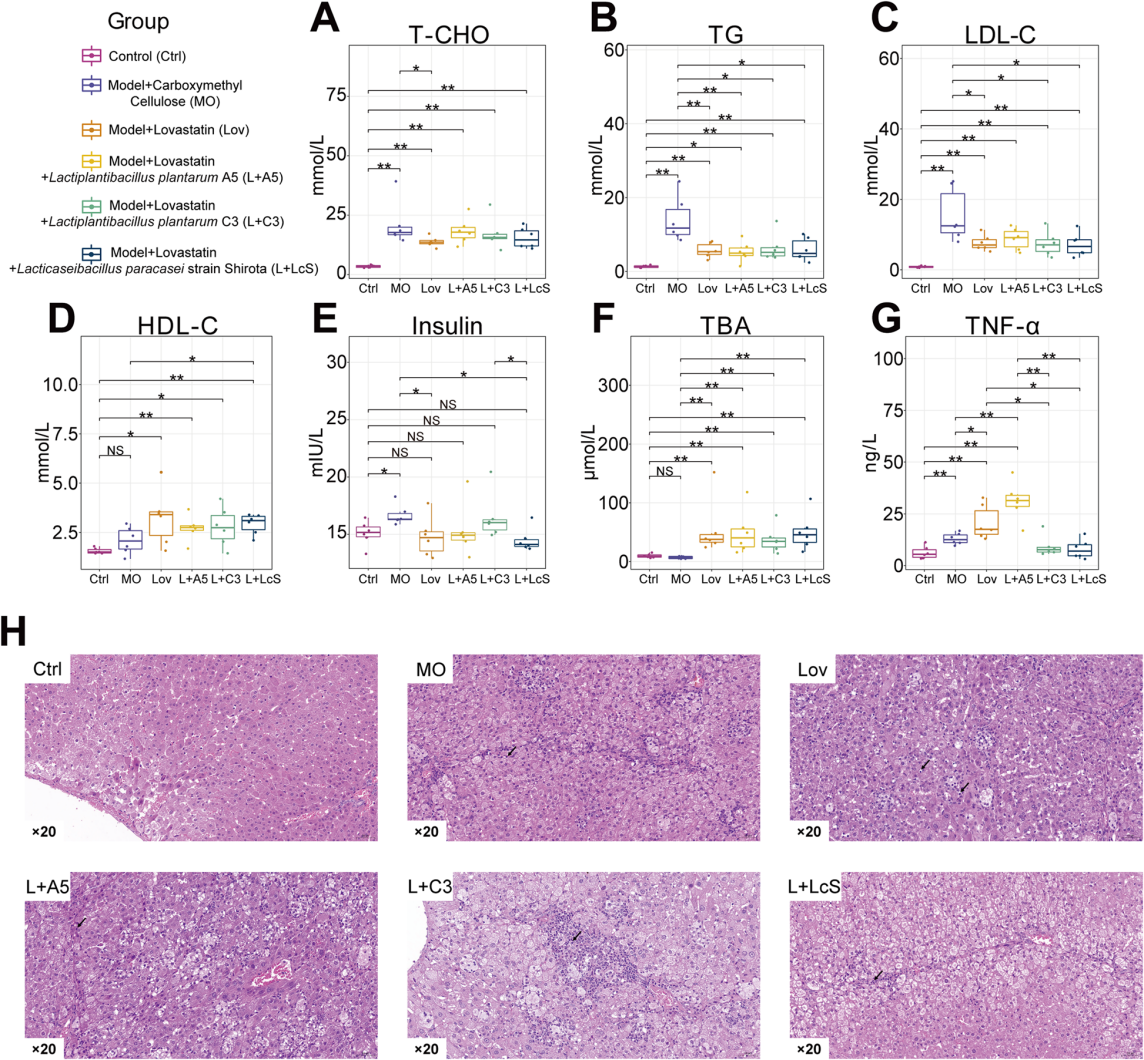
Synergistic intake of probiotics didn’t affect the therapeutic effect of lovastatin on hyperlipidemia, but was conducive to liver protection in vivo. **A–G** Serum T-CHO, TG, LDL-C, HDL-C, insulin, TBA, and TNF-α concentrations measured by ELISA (Wilcoxon rank-sum test, *p < 0.05, **p < 0.01).** H** Representative histological sections stained by H&E of liver tissue at 20 × magnification

To study whether probiotics can accelerate the consumption of lovastatin in the host intestine and affect the efficacy, the golden hamster, which has liver and lipid metabolic characteristics closest to those of humans, was selected. The golden hamster model of mixed hyperlipidemia was constructed, and when the model was successfully established (Supplemental Fig. 2), animals were treated with lovastatin alone (Lov), lovastatin with *Lactiplantibacillus plantarum* A5 (L + A5), lovastatin with *Lactiplantibacillus plantarum* C3 (L + C3), lovastatin with *Lacticaseibacillus paracasei* strain Shirota (L + LcS), and no drug treatment (MO) (*n* = 6) (Fig. 2). The difference in the treatment effect was comprehensively evaluated by the following blood indexes: total cholesterol (T-CHO), triglycerides (TG), low-density lipoprotein cholesterol (LDL-C), high-density lipoprotein cholesterol (HDL-C), and insulin. The results showed that although the Lov group had significantly reduced T-CHO (*p* < 0.05), there was no significant difference in the T-CHO content between the Lov group and the three probiotic groups (Fig. 3A). The Lov group and the three probiotic groups had significantly reduced TG (*p* < 0.05) (Fig. 3B). At the same time, except for the L + A5 group, the LDL-C of the other three groups that received lovastatin was decreased significantly (*p* < 0.05), but there was no difference among the four groups that also received lovastatin (Fig. 3C). In addition, only the L + LcS group had a significant increase in HDL-C (*p* < 0.05), and there was no significant change in the other groups (Fig. 3D). Subsequently, the insulin results showed that both the Lov group and the L + LcS group had significantly reduced insulin contents (*p* < 0.05). However, there was no difference in the insulin content among the Lov group and the three probiotic groups (Fig. 3E). The above results suggested that although the therapeutic effect of each blood index was slightly different, there was no significant difference between the therapeutic effect of lovastatin alone and that of probiotics.

It is worth noting that total bile acids (TBAs) in the blood showed that the intake of lovastatin significantly increased total bile acids compared with no treatment (*p* < 0.01) (Fig. 3F). At the same time, tumor necrosis factor-α (TNF-α) in the Lov group was significantly higher than that in the MO group, L + C3 group, and L + LcS group (*p* < 0.05); TNF-α in the L + A5 group was significantly higher than that in the MO group, L + C3 group, and L + LcS group (*p* < 0.01). However, the Lov group and L + A5 group showed no significant difference in the TNF- α content (Fig. 3G). The results of liver tissue observation showed that the degree of liver inflammatory cell infiltration in the L + LcS group was significantly less than that in the other model groups. The liver inflammatory cell infiltration was relatively serious in the Lov group (Fig. 3H). This finding showed that lovastatin intake makes the liver prone to inflammatory reactions, but the intake of probiotics could slow the inflammation to some extent.

### Probiotics improved the systemic disorder in the gut microbiota and metabolites induced by lovastatin consumption

**Fig. 4:**
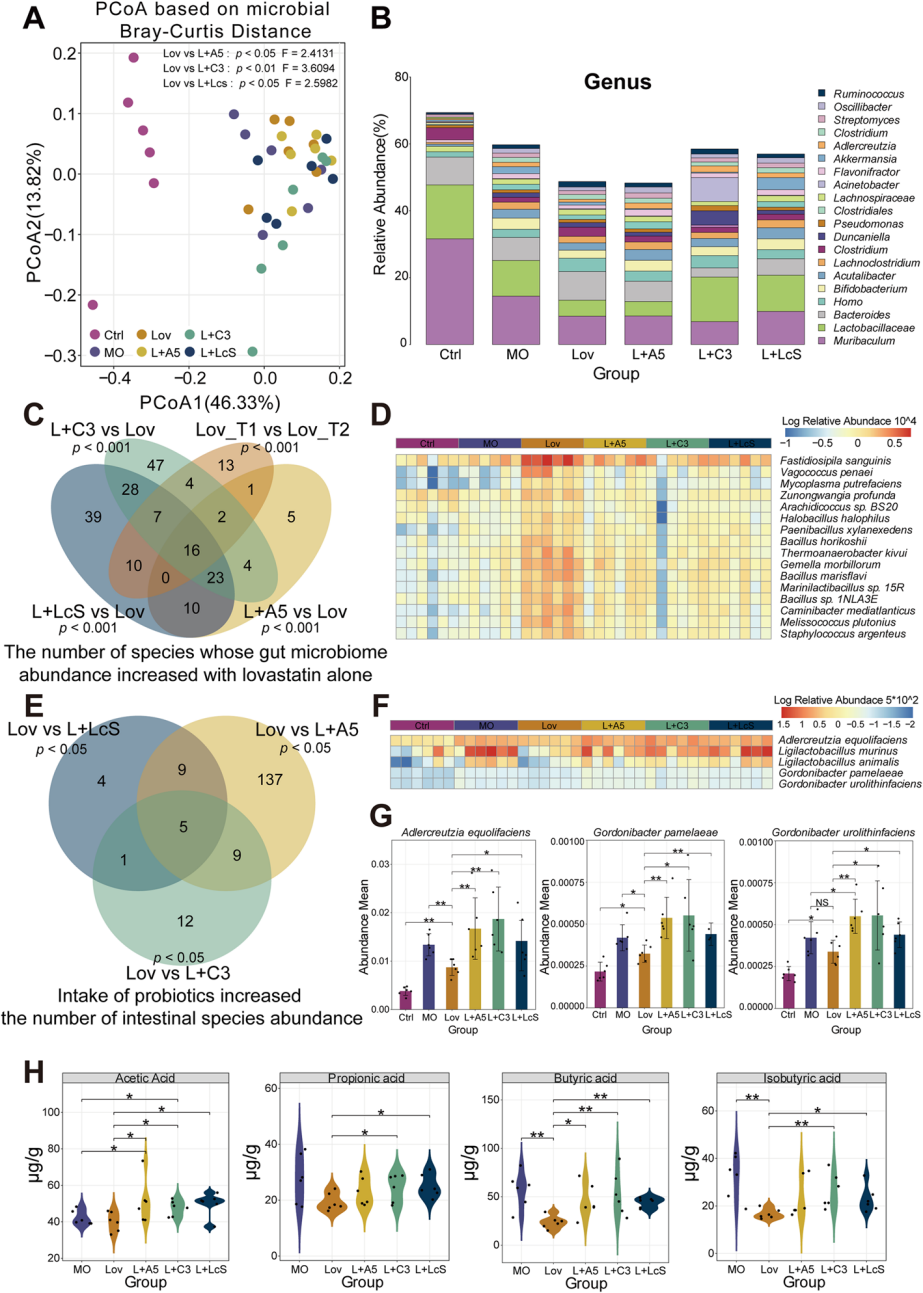
Synergistic intake of probiotics can improve the intestinal microbiota and metabolite disorders caused by lovastatin intake. **A** Principal coordinate analysis (PCoA, Bray–Curtis) of gut microbiota from each group of golden hamsters. The *p* value represented the significance between the two groups (Wilcoxon rank-sum test). **B** Identification of fecal microbial composition at genus level. **C** There was a co-significant increase of 16 intestinal microbiota in lovastatin alone compared with lovastatin in combination with probiotics and before lovastatin. The *p* value represented the significance between the two groups (Wilcoxon rank-sum test). **D** The Lov group significantly increased the abundance of 16 intestinal microbes compared with other treatment groups. **E** The three lovastatin synergistic probiotics treatment groups significantly increased five gut microbiota compared with the lovastatin alone treatment group. **F** The abundance of 5 microbes in the three lovastatin synergistic probiotics treatment groups and lovastatin group increased significantly. **G** Among the five significantly increased microbiota, three represent the abundance of microbiota (*Adlercreutzia equolifaciens*, *Gordonibacter pamelaeae*, and *Gordonibacter urolithinfaciens*) (Wilcoxon rank-sum test, *p < 0.05, **p < 0.01). **H** At the last time point (week 12), the quantitative concentration of SCFAs in the intestinal contents of golden hamsters. Significant differences were evaluated by Wilcoxon test (*p < 0.05; **p < 0.01)

To study the potential role of probiotics in lovastatin treatment and the reduction of liver inflammation through the regulation of the gut microbiome in hyperlipidemic hamsters, we conducted metagenomics analysis at multiple time points. Principal coordinate analysis (PCoA) of the Bray–Curtis distance was used to compare the composition of the gut microbiota in each experimental group. Compared with the Lov group, the microbial structure of the three probiotic groups was changed (Adonis test, *p* < 0.05) (Fig. 4A). The microbial composition of each group was further analyzed. For the four groups treated with lovastatin, the genus levels of *Lactobacillaceae* in the Lov group and the L + A5 group were significantly lower than those in the L + C3 group and L + LcS group. The content of *Bacteroides* in the Lov group was the highest (Fig. 4B). *Lactobacillaceae* plays an important role in regulating intestinal microbes and enhancing host immunity, while *Bacteroides* is prone to endogenous infection.

The genus-level results seem to suggest that lovastatin intake has a harmful effect on the host gut microbiome. By going deep into the species-level structure, 53 intestinal microorganisms (*p* < 0.01) were significantly reduced in the Lov group after 4 weeks of intake. A total of 16 microorganisms (Fig. 4C) were screened from the intersection of the strains that were significantly reduced (*p* < 0.01) compared with the Lov group and the three probiotic groups. These strains were *Fastidiosipila sanguinis*, *Vagococcus penaei*, *Mycoplasma putrefaciens*, *Zunongwangia profunda*, *Arachidicoccus* sp. BS20, *Halobacillus halophilus*, *Paenibacillus xylanexedens*, *Bacillus horikoshii*, *Thermoanaerobacter kivui*, *Gemella morbillorum*, *Bacillus mariflavi*, *Marinilactibacillus* sp. 15R, *Bacillus* sp. 1NLA3E, *Caminibacter mediatlanticus, Melissococcus plutonius*, and *Staphylococcus argenteus*. Most of them are harmful microorganisms (Fig. 4D). Next, five strains (Fig. 4E) were found that were significantly increased (*p* < 0.05) in the L + A5 group, L + C3 group, and L + LcS group compared with the Lov group. They were *Adlercreutzia equolifaciens*, *Ligilactobacillus murinus, Ligilactobacillus animalis*, *Gordonibacter pamelaeae*, and *Gordonibacter urolithinfaciens*. Surprisingly, these five intestinal microbiota are host beneficial bacteria (Fig. 4F). In particular, *Adlercreutzia equolifaciens* has a certain connection with liver diseases and is a “barometer” of liver health. In addition, *Gordonibacter pamelaeae* and *Gordonibacter urolithinfaciens* can provide urolithin, which can affect cell lipid metabolism and fat production, and prevent fat accumulation caused by diet (Fig. 4G). The above results suggest that the intake of lovastatin is harmful to the intestinal microbes of the host, but when combined with the intake of probiotics, the intestinal beneficial bacteria will increase and the intestinal microbiota will be improved.

We qualitatively and quantitatively identified acetic acid, propionic acid, butyric acid, and isobutyric acid in hamster intestinal contents. The results showed that the contents of acetic acid and butyric acid in the Lov group were significantly lower than those in the three probiotic groups (*p* < 0.05). Moreover, the contents of propionic acid and isobutyric acid in the L + C3 group and L + LcS group were significantly higher than those in the Lov group (*p* < 0.05). This finding may indicate that probiotics administered by gavage can improve the accumulation of short-chain fatty acids (SCFAs) in hamsters (Fig. 4H).

### The synergetic effects of lovastatin and probiotics on liver transcription

**Fig. 5:**
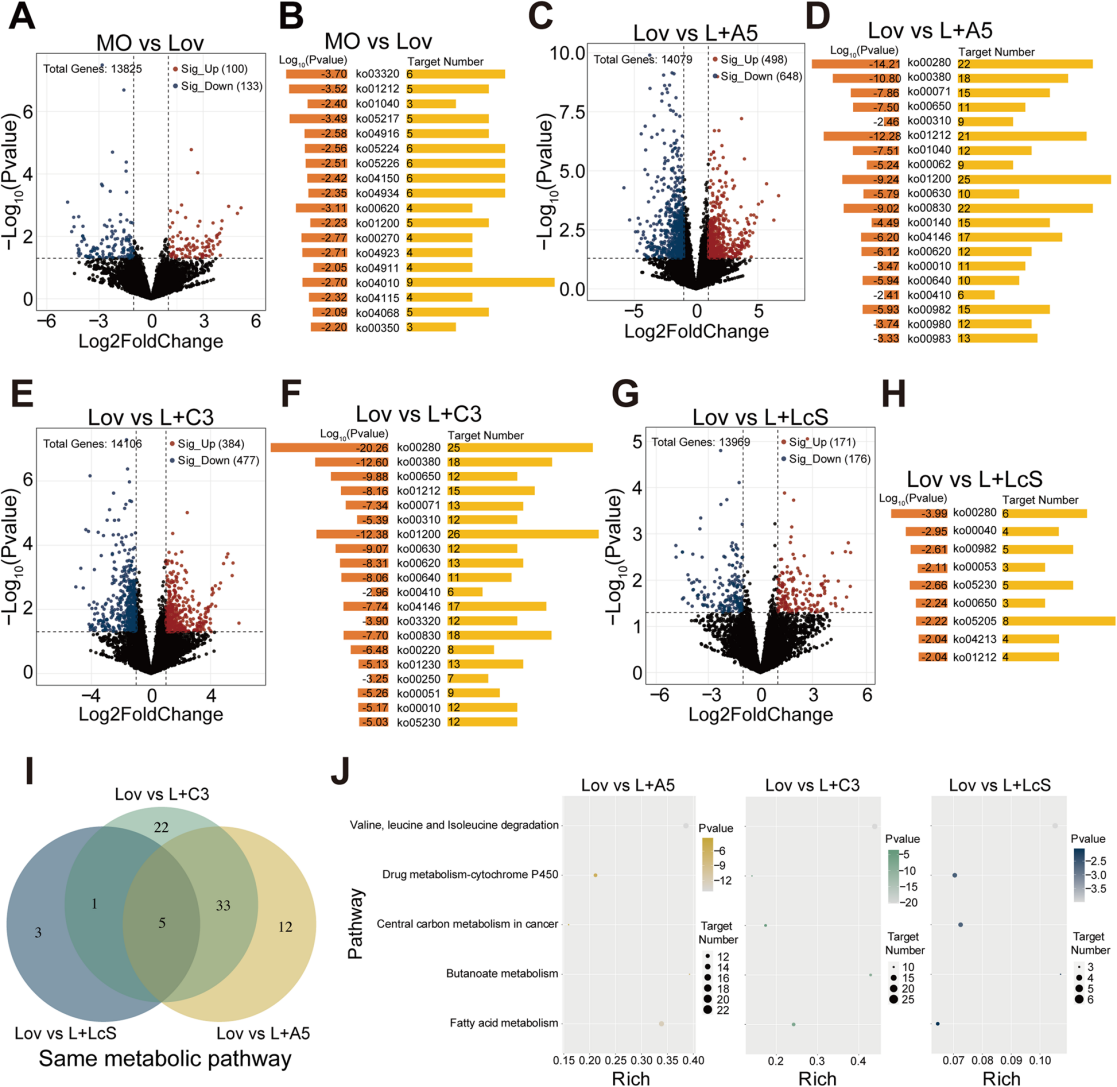
Differentially expressed genes and KEGG enrichment analysis. **A** Volcano plots of all gene comparisons between the MO group and Lov group. Red and blue dots indicated that when *p* value was less than 0.05 and Log2FoldChange value was greater than 1 or less than − 1, they were significantly upregulated and downregulated differentially expressed genes (DEGs). **B** Metascape analysis showed that the 18 clusters enriched KEGG pathway were representative in the list of upregulated and downregulated genes. Each line represented a KEGG ontology. Left bar: Log (*p* value) indicated a significant pathway. The right bar indicated the number of genes enriched in the pathway (hypergeometric test, **p < 0.01). **C** Volcano plots of all gene comparisons between Lov group and L + A5 group. **D** Metascape analysis showed that the top 20 clusters enriched KEGG pathway were representative in the list of upregulated and downregulated genes. **E** Volcano plots of all gene comparisons between the Lov group and L + C3 group. **F** Metascape analysis showed that the top 20 clusters enriched KEGG pathway were representative in the list of upregulated and downregulated genes. **G** Volcano plots of all gene comparisons between the Lov group and L + LcS group. **H** Metascape analysis showed that the 9 clusters enriched KEGG pathway were representative in the list of upregulated and downregulated genes. **I** Compared with lovastatin group, the three probiotics groups were enriched in five metabolic pathways. **J** The five metabolic pathways enriched by the three probiotics groups were valine, leucine, and isoleucine degradation; drug metabolism-cytochrome P450; central carbon metabolism in cancer; butanoate metabolism; and fatty acid metabolism

Because lovastatin acts on the liver, to determine the changes in host metabolic pathways caused by lovastatin-induced inflammation and probiotic treatment, we analyzed the transcriptional response of liver tissue by high-throughput sequencing. Compared with the MO group, 133 genes were downregulated and 100 genes were upregulated in the Lov group (Fig. 5A). These differentially annotated genes mainly came from 18 metabolic pathways. The expression of the Me1, Scd1, Scd4, and Acsl5 genes was downregulated in the PPAR signaling pathway (ko03320), resulting in a reduction in lipogenesis controlled by the skeletal muscle. The downregulation of the Sgk1, Wnt1, Wnt11, and Wnt9a genes in the mTOR signaling pathway (ko04150) indicates that cell survival decreases in the insulin signaling pathway. In addition, the downregulation of Adcy8, Slc2a2, and Adcy5 and the upregulation of Trpm4 in the insulin secretion pathway may lead to decreased insulin secretion (Fig. 5B). These pathway analysis results may indicate that lovastatin intake can effectively alleviate hyperlipidemia and reduce obesity-related indicators.

Compared with the Lov group, 648 genes were downregulated, and 498 genes were upregulated in the L + A5 group (Fig. 5C). These differentially annotated genes were enriched in 50 metabolic pathways, and only the top 20 metabolic pathways (Fig. 5D) are shown in the figure. Subsequently, compared with those of the Lov group, 477 genes were downregulated and 384 genes were upregulated in the L + C3 group (Fig. 5E), and a total of 61 metabolic pathways were enriched. Only the top 20 metabolic pathways (Fig. 5F) are shown in the figure. In addition, 176 genes were downregulated and 171 genes were upregulated in the L + LcS group compared with the Lov group (Fig. 5G). These differentially annotated genes mainly came from nine metabolic pathways (Fig. 5H). The metabolic pathways enriched by the three probiotic groups and the Lov group intersected, and five coenriched metabolic pathways were obtained (Fig. 5I). They are valine, leucine, and isoleucine degradation (ko00280); drug metabolism-cytochrome P450 (ko00982); central carbon metabolism in cancer (ko05230); butanoate metabolism (ko00650); and fatty acid metabolism (ko01212) (Fig. 5J). It is worth noting that the genes enriched in the valine, leucine, and isoleucine degradation pathways were downregulated, which means that valine, leucine, and isoleucine degradation decreased in the three probiotic groups, and the relative contents of valine, leucine, and isoleucine increased in vivo. Isoleucine, leucine, and valine work together to repair muscles, control blood sugar, and provide energy to body tissues.

### Potential mechanism by which probiotics alleviate the lovastatin-induced liver inflammatory response

**Fig. 6:**
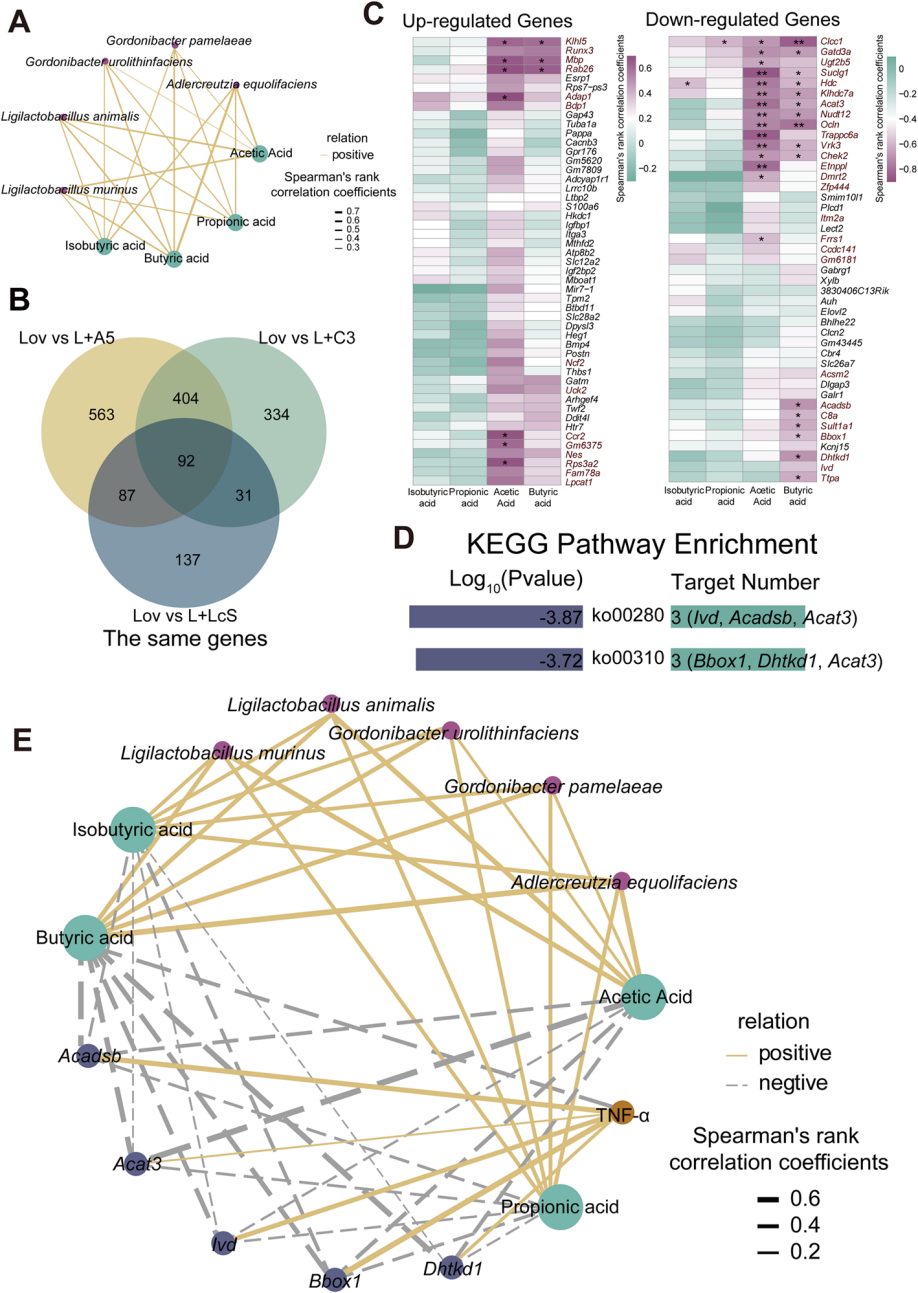
Probiotics increased the SCFAs produced by microbes, and SCFAs could regulate the expression of liver genes. **A** The five intestinal bacteria increased by the three probiotics groups were positively correlated with acetic acid, propionic acid, butyric acid, and isobutyric acid (*r* > 0.5). The thickness of the solid line indicates the strength of the correlation. **B** Comparing the L + A5 group with the Lov group, the L + C3 group with the Lov group, and the L + LcS group with the Lov group, there were 92 identical differentially expressed genes (|Log2FoldChange|≥ 1, *p* < 0.05). **C** Correlation between 92 differential genes (upregulated genes and downregulated genes) and SCFAs. Genes in red: | *r* |> 0.5. (Wilcoxon rank-sum test, *p < 0.05, **p < 0.01). **D** Differential genes with strong correlation (| *r* |> 0.5) with SCFAs were enriched into two metabolic pathways. **E** Correlation between liver expressed genes, fecal SCFAs, and intestinal microbiota. The solid line indicated positive correlation, and the dotted line indicated negative correlation. The thickness of the line indicated the strength of the correlation

The differences in the intestinal environment and liver transcription between probiotics-treated and untreated golden hamsters treated with lovastatin prompted us to speculate whether there was a link between the two. To clarify this relationship, we first performed Spearman’s correlation analysis on the microbiota and SCFAs jointly upregulated by the three probiotic groups and found that the five upregulated bacteria were moderately positively correlated with butyric acid (*r* > 0.5, *p* < 0.01), and *Ligilactobacillus murinus* and *Ligilactobacillus animalis* were moderately positively correlated with acetic acid (*r* > 0.5, *p* < 0.01) (Fig. 6A). Subsequently, we found 92 common differentially expressed genes between the Lov group and the L + A5 group, the Lov group and the L + C3 group, and the Lov group and the L + LcS group (Fig. 6B). Among them, 49 genes were upregulated, and 43 genes were downregulated. SCFAs can regulate gene expression. We performed Spearman’s correlation analysis on SCFAs and 49 upregulated genes and 43 downregulated genes. Among them, there were 14 upregulated genes that were moderately related to SCFAs (*r* > 0.5), which were positively correlated, and 27 downregulated genes that were moderately related to SCFAs (*r* < 0.5), which were negatively correlated (Fig. 6C). These genes related to SCFAs were enriched in KEGG pathways. Five genes were enriched in two pathways (*p* < 0.01, min enrichment = 1.5), namely, valine, leucine, and isoleucine degradation (ko00280) and lysine degradation (ko00310) (Fig. 6D).

**Fig. 7:**
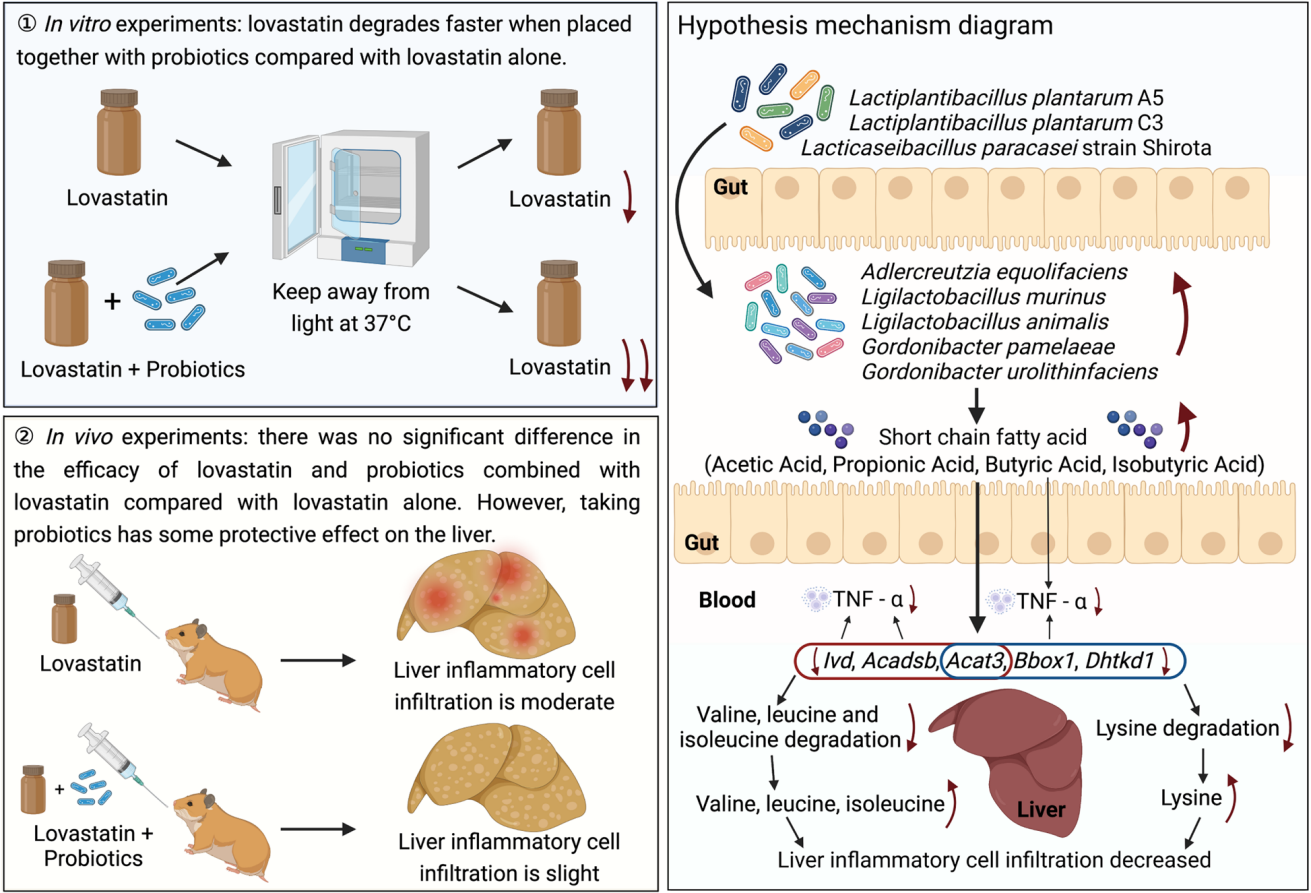
Experimental schematic diagram and potential mechanism diagram of in vivo results. (A) Left: schematic diagram of the results of in vitro and in vivo experiments. Right: schematic diagram of probiotic supplement alleviating liver inflammatory response. Black arrows indicated promotion. The red arrow next to the corresponding text indicated whether it is up or down

Then, we further explained the potential relationship between the intestinal environment and host immunity from the overall correlation between the probiotic-upregulated microbiome, SCFAs, differential genes, and immunity. The results showed that SCFAs regulated differential genes in the liver, which was negatively correlated with TNF-α, and this was related to the microbial species that were upregulated in the probiotic groups (Fig. 6E). Therefore, we speculated that probiotics can reduce the body’s inflammatory response and improve the mechanism of liver protection. Probiotics regulate the intestinal microbes of golden hamsters by increasing beneficial bacteria and promote the accumulation of acetic acid, propionic acid, butyric acid, and isobutyric acid in the intestine. These SCFAs that are produced can further downregulate some genes transcribed by the liver, especially *Ivd*, *Acadsb*, *Acat3*, *Bbox1*, and *Dhtkd1*. Among them, *Ivd*, *Acadsb*, and *Acat3* are enriched in the valine, leucine, and isoleucine degradation pathway, which increases the relative content of valine, leucine, and isoleucine in vivo; *Acat3*, *Bbox1*, and *Dhtkd1* are enriched in the lysine degradation pathway, which increases the relative content of lysine in vivo. Four essential amino acids work together in the body to protect the liver and reduce liver inflammation. At the same time, butyric acid, together with the *Ivd*, *Acadsb* and *Bbox1* genes, reduced the serum TNF-α content and the inflammatory response (Fig. 7A).

## Discussion

Studies have shown that the microbiota can change the activity and efficacy of drugs through chemical transformation, and probiotics that are beneficial to the health of the host are no exception; but sometimes, this change may not be wanted, and it may affect the efficacy. Lovastatin is a very popular lipid-lowering drug in the clinic [23]. It can reduce the synthesis of cholesterol [24] and increase the synthesis of low-density lipoprotein receptor [25]. At the same time, it can also reduce the level of serum triglycerides and increase the level of blood high-density lipoprotein [24]. Lovastatin’s main action site is the liver. Lovastatin can work only after the liver hydrolyses it into an active hydroxy acid. Therefore, we suspected that if lovastatin is taken with probiotics, the presence of probiotics will cause lovastatin to be degraded into lovastatin hydroxy acid in the intestine in advance, thereby reducing the content reaching the liver and affecting the efficacy. Based on this conjecture, we conducted in vitro and in vivo experiments on the effect of probiotics on the efficacy of lovastatin.

In the in vitro experiments, we used *Lactiplantibacillus plantarum*, the most common lactic acid bacteria in fermented food, and *Lacticaseibacillus paracasei* strain Shirota, the “star strain” in milk drinks, as the research objects. The degradation degree of lovastatin by 83 strains of *Lactiplantibacillus plantarum* from the self-built bacterial bank and the *Lacticaseibacillus paracasei* strain Shirota was determined by HPLC. It was found that probiotics could degrade lovastatin to a certain extent. We selected *Lactiplantibacillus plantarum* A5 with the strongest ability to degrade lovastatin, *Lactiplantibacillus plantarum* C3 with the weakest ability to degrade lovastatin and *Lacticaseibacillus paracasei* strain Shirota as representative probiotics for in-depth study. To clarify the possible reasons for the degradation of lovastatin by probiotics, we monitored the transcription of three probiotics under the synergistic effect of lovastatin and found that the Rn18s gene expression of the three probiotics was significantly downregulated under the effect of lovastatin. Rn18s is a mitochondrial functional gene [26].

Subsequently, we conducted in vivo experiments using three representative probiotics to determine whether the degradation of lovastatin by probiotics occurs in the body, thereby affecting the efficacy. However, the presence of probiotics would have not a significant effect on the effects of lovastatin on blood indicators such as T-CHO, TG, LDL-C, HDL-C, and insulin. This means that the synergistic intake of probiotics will not affect the efficacy of lovastatin (The relief of hyperlipidemia in golden hamsters is mainly due to lovastatin, and probiotic therapy alone cannot significantly alleviate the occurrence of hyperlipidemia in golden hamsters, Supplemental Fig. 3). At the same time, liver tissue section data showed that the intake of *Lacticaseibacillus paracasei* strain Shirota could significantly reduce liver inflammatory cell infiltration. These data also showed that the introduction of probiotics can alleviate liver inflammation to a certain extent.

We observed the changes in intestinal microbes after lovastatin and probiotics were used together and were surprised to find that compared with golden hamsters treated with lovastatin alone, the intake of probiotics increased the content of *Lactobacillaceae. Lactobacillaceae* [27] has the functions of regulating intestinal microbiota, enhancing immunity, protecting gastric mucosa, improving intestinal function, defecating, preventing and treating diarrhea, and promoting digestion, as well as antitumor and antioxidant properties. At the same time, an in-depth study of the levels of intestinal species in the probiotic groups showed that lovastatin treatment alone led to a significant increase in the number of conditional pathogens in the intestine that produce inflammation, such as *Gemella morbilorum* [28], which may be related to the side effects of drug treatment. At the same time, we found that the contents of *Adlercreutzia equolifaciens*, *Ligilactobacillus murinus*, *Ligilactobacillus animalis*, *Gordonibacter pamelaeae*, and *Gordonibacter urolithinfaciens* in the intestinal tract of the three probiotic groups were significantly higher than those of the lovastatin treatment alone group. Studies have shown that lovastatin intake has certain harmful effects on the gastrointestinal tract, liver, and muscle. However, the significantly increased strains in the probiotic groups are closely related to the alleviation of the side effects of lovastatin. *Adlercreutzia equolifaciens* [29] is not only closely related to the health of the liver but can also reduce inflammation, reduce blood glucose, and limit weight gain. Urolithin produced by *Gordonibacter pamelaeae* and *Gordonibacter urolithinfaciens* [30] can not only induce brown fat activation and white fat browning and reduce fat accumulation but can also reverse muscle decline and protect host muscle. Subsequently, compared with lovastatin alone, we found that SCFAs represent beneficial intestinal microbial metabolites that were significantly increased in the probiotic groups. Liver transcriptome data also showed that compared with lovastatin alone, the valine, leucine, and isoleucine degradation pathways in the probiotic groups were downregulated, which could increase the content of the essential amino acids valine, leucine, and isoleucine in the body. Studies have shown that appropriate amounts of valine, leucine, and isoleucine can repair muscles, help burn visceral fat, and protect the liver [31].

In addition, we also explored the potential mechanism by which probiotics slow inflammation through the regulatory relationship between the microbiome and liver transcription. We found that more acetic acid, propionic acid, butyric acid, and isobutyric acid accumulated in the colon feces of golden hamsters treated with probiotics. These SCFAs have a strong positive correlation with the increase in beneficial bacteria in the probiotics groups. Therefore, we can infer that probiotics can increase the production of SCFAs by increasing the colonization of specific beneficial bacteria in the intestine of golden hamsters. Subsequently, SCFAs enter the portal vein circulation of the liver through the basolateral membrane, resulting in the downregulation of the expression of the genes *Ivd*, *Acadsb*, *Acat3*, *Bbox1*, and *Dhtkd1*, thereby decreasing the valine, leucine, and isoleucine degradation and lysine degradation pathways. The decrease in the pathways leads to an increase in the relative contents of the essential amino acids valine, leucine, isoleucine, and lysine in the host, which plays a protective role in alleviating the inflammatory response of the host’s body and liver. The four essential amino acids are beneficial amino acids that can enhance the body’s immunity and anti-virus activity, promote fat oxidation, protect muscle and liver, and have other positive nutritional significance. Since essential amino acids refers to amino acids that cannot be synthesized by the human body or other vertebrates or whose synthesis speed is far from meeting the needs of the body and must be supplied by food protein, when the diet is the same, the essential amino acids produced by the body of each group are relatively similar. Recent studies have reported that excessive branched-chain amino acids are harmful to human health [32]. However, in this study, the branched-chain amino acids contents in the probiotic groups were relatively low compared with those in the Lov group alone, so they are beneficial to human health. At the same time, a small amount of SCFAs produced by the colon reaches the systemic circulation to reduce the content of TNF-α in the blood, alleviate the blood inflammatory reaction, and indirectly alleviate liver inflammation [33].

We evaluated the relationship between probiotics and drugs from a new perspective and assessed the possible impact of probiotics on drugs from two different perspectives. It was found that probiotics that cause drug degradation in vitro will not have a significant impact on the therapeutic effect of drugs in vivo. In addition, the presence of probiotics may alleviate the side effects of drugs. However, this study has the following two limitations. First, due to various reasons, such as the number of experimental groups, the sample size of each experimental group was small (*n* = 6). Second, we did not verify the mechanism after proposing the potential mechanism. Regarding the potential mechanism of this study, we can use liver gene knockout technology for verification, but liver gene knockout technology is not currently popular, and it is difficult to perform.

## Conclusions

The supplementation of probiotics produced beneficial metabolites, including acetic acid and butyric acid, in the intestine by promoting beneficial microbes (*Adlercreutzia equolifaciens*, *Ligilactobacillus murinus*, *Ligilactobacillus animalis*, *Gordonibacter pamelaeae*, and *Gordonibacter urolithinfaciens*). Intestinal metabolites affected the expression of the liver *Ivd*, *Acadsb*, *Acat3*, *Bbox1*, and *Dhtkd1* genes through the gut-liver axis; increased the relative content of the essential amino acids valine, leucine, isoleucine, and lysine; and finally improved the liver inflammatory response of the host. This study aims to reveal the impact of probiotics on the human body from a unique perspective, suggesting the impact of taking probiotics while taking drugs.

### Supplementary Information

**Additional file 1: Fig. S1.** The detailed procedure of in vitro experiment. **Fig. S2.** The golden hamster model of mixed hyperlipidemia. **Fig. S3.** The relief of hyperlipidemia in golden hamsters is mainly due to lovastatin, and probiotic therapy alone cannot significantly alleviate the occurrence of hyperlipidemia in golden hamsters.

**Additional file 2.**

## Data Availability

The metagenome data in this study have been deposited in BioProject database under the BioProject ID of PRJNA862662 and PRJNA862692.
