# Supplementary figures and images for: Understanding the “individual drug reaction” from the perspective of the interaction between probiotics and lovastatin in vitro and in vivo
Source: Microbiome. 2023 Sep 25;11:209. doi: 10.1186/s40168-023-01658-z (PMC10518969; doi:10.1186/s40168-023-01658-z)

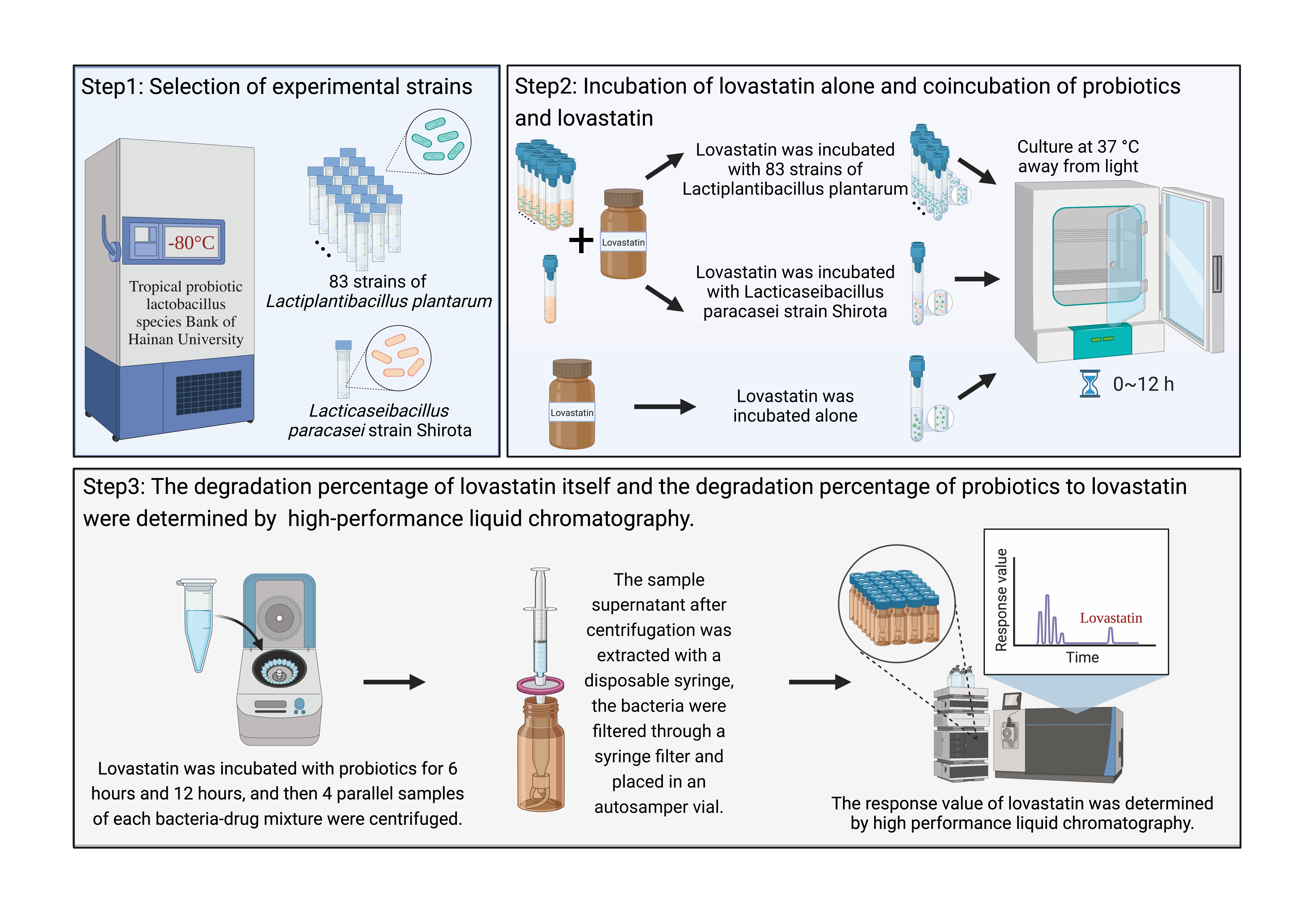

Supplement: Supplementary file 2 — Additional file 1: Fig. S1. The detailed procedure of in vitro experiment. Fig. S2. The golden hamster model of mixed hyperlipidemia. Fig. S3. The relief of hyperlipidemia in golden hamsters is mainly due to lovastatin, and probiotic therapy alone cannot significantly alleviate the occurrence of hyperlipidemia in golden hamsters. [file 40168_2023_1658_MOESM1_ESM.zip › Supplemental figure 1.tif]

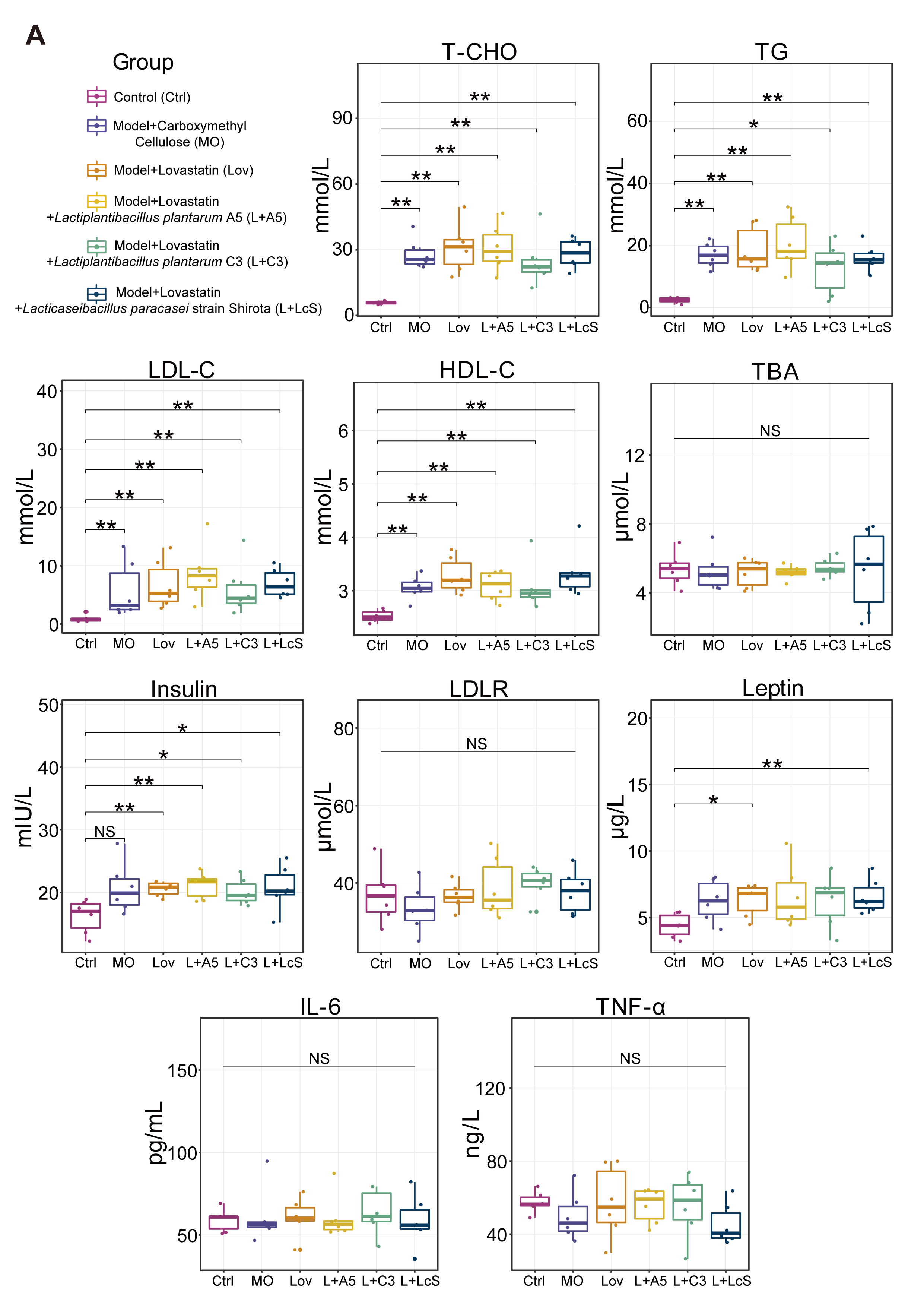

Supplement: Supplementary file 2 — Additional file 1: Fig. S1. The detailed procedure of in vitro experiment. Fig. S2. The golden hamster model of mixed hyperlipidemia. Fig. S3. The relief of hyperlipidemia in golden hamsters is mainly due to lovastatin, and probiotic therapy alone cannot significantly alleviate the occurrence of hyperlipidemia in golden hamsters. [file 40168_2023_1658_MOESM1_ESM.zip › Supplemental figure 2.tif]

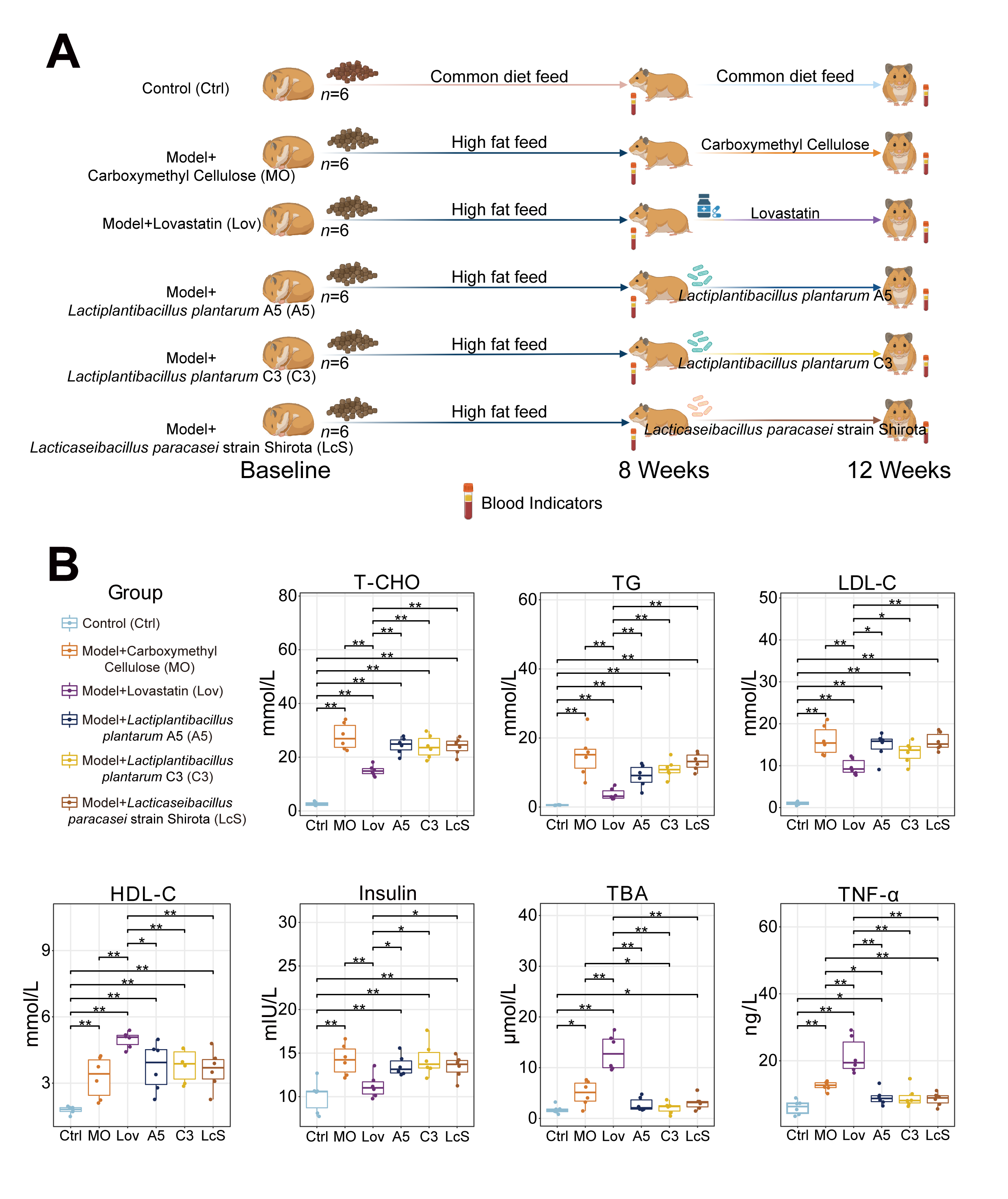

Supplement: Supplementary file 2 — Additional file 1: Fig. S1. The detailed procedure of in vitro experiment. Fig. S2. The golden hamster model of mixed hyperlipidemia. Fig. S3. The relief of hyperlipidemia in golden hamsters is mainly due to lovastatin, and probiotic therapy alone cannot significantly alleviate the occurrence of hyperlipidemia in golden hamsters. [file 40168_2023_1658_MOESM1_ESM.zip › Supplemental figure 3.tif]
